# Perceived social risk in medical decision-making for physical child abuse: a mixed-methods study

**DOI:** 10.1186/s12887-017-0969-7

**Published:** 2017-12-22

**Authors:** Heather T. Keenan, Kristine A. Campbell, Kent Page, Lawrence J. Cook, Tyler Bardsley, Lenora M. Olson

**Affiliations:** 1 Division of Pediatric Critical Care, Department of Pediatrics, University of Utah School of Medicine, P.O. Box 581289, Salt Lake City, UT 84158 USA; 2 Division of Child Protection and Family Health, University of Utah School of Medicine, P.O. Box 581289, Salt Lake City, UT 84158 USA; 3 Department of Pediatrics, University of Utah School of Medicine, P.O. Box 581289, Salt Lake City, UT 84158 USA

**Keywords:** Child abuse pediatrics, Bias, Disparity

## Abstract

**Background:**

The medical literature reports differential decision-making for children with suspected physical abuse based on race and socioeconomic status. Differential evaluation may be related to differences of risk indicators in these populations or differences in physicians’ perceptions of abuse risk. Our objective was to understand the contribution of the child’s social ecology to child abuse pediatricians’ perception of abuse risk and to test whether risk perception influences diagnostic decision-making.

**Methods:**

Thirty-two child abuse pediatrician participants prospectively contributed 746 consultations from for children referred for physical abuse evaluation (2009–2013). Participants entered consultations to a web-based interface. Participants noted their perception of child race, family SES, abuse diagnosis. Participants rated their perception of social risk for abuse and diagnostic certainty on a 1–100 scale. Consultations (*n* = 730) meeting inclusion criteria were qualitatively analyzed for social risk indicators, social and non-social cues. Using a linear mixed-effects model, we examined the associations of social risk indicators with participant social risk perception. We reversed social risk indicators in 102 cases whilst leaving all injury mechanism and medical information unchanged. Participants reviewed these reversed cases and recorded their social risk perception, diagnosis and diagnostic certainty.

**Results:**

After adjustment for physician characteristics and social risk indicators, social risk perception was highest in the poorest non-minority families (24.9 points, 95%CI: 19.2, 30.6) and minority families (17.9 points, 95%CI, 12.8, 23.0). Diagnostic certainty and perceived social risk were associated: certainty increased as social risk perception increased (Spearman correlation 0.21, *p* < 0.001) in probable abuse cases; certainty decreased as risk perception increased (Spearman correlation (−)0.19, *p* = 0.003) in probable not abuse cases. Diagnostic decisions changed in 40% of cases when social risk indicators were reversed.

**Conclusions:**

CAP risk perception that poverty is associated with higher abuse risk may explain documented race and class disparities in the medical evaluation and diagnosis of suspected child physical abuse. Social risk perception may act by influencing CAP certainty in their diagnosis.

## Background

Medical decision-making in cases of suspected child abuse has important consequences. An incorrect diagnosis may return an abused child to the abusive home; conversely, it may subject non-abusive families to legal remedies and stigma. Prior studies have shown that physicians are more comfortable reporting poor children for abuse [1]; African American children are more likely than Caucasian children to undergo an abuse evaluation for fractures [2]; Caucasian children are less likely to be evaluated for abusive head trauma than minority children [3]; and decisions about discharging children home from the emergency department with a diagnosis of abuse depends upon socioeconomic status [4]. These studies documented differential decision-making in the evaluation and disposition of children with suspected physical abuse.

Differences in medical decision-making may reflect differences in risk indicators for abuse among poor and minority children not captured in administrative datasets. They may also reflect differences in treating physicians’ perceptions of the risk for abuse among poor and minority children. Social risk perception, defined here as the perceived risk for child abuse based on social aspects of the child’s medical evaluation rather than physical, laboratory, or radiologic aspects of the evaluation, may play a role in how children are evaluated. Medical assessments of child physical abuse risk may be particularly vulnerable to physicians’ perceptions of abuse risk as child abuse pediatricians (CAPs) incorporate the child’s entire social ecology into their evaluation [5]. Prior studies have not identified information important to decision making beyond socioeconomic status (SES) and race that may influence physicians’ perceptions of the child’s social risk for abuse.

The goals of this study were twofold: to define factors that CAPs incorporate into their social risk perception of physical child abuse when evaluating an injured child and to understand whether self-rated social risk perception influences the diagnosis of abuse. We hypothesized that CAPs would incorporate family characteristics other than published risk indicators into their perceptions of social risk and that risk perception would be associated with diagnostic decision-making.

## Methods

### Study context

For this mixed methods study, we collected inpatient medical consultation notes by CAPs for three types of injury cases referred for child physical abuse consultation from 2009 to 2013. Injury types included neurotrauma, long bone fracture and skull fracture. These injury types were chosen as the mechanism of injury may be abuse or non-abuse and these injury types are commonly evaluated by CAP. The study was approved by Institutional Review Board for the University of Utah and each participant’s institution. A Certificate of Confidentiality was obtained from the National Institute of Child Health and Human Development.

### Participants

Thirty-two CAPs were recruited from two, professional physician child maltreatment groups in the United States: the Ray E. Helfer Society and the American Academy of Pediatrics, Section on Child Abuse and Neglect. CAP participants were required to have 5 years in pediatric practice post-residency, pediatric board certification, spend at least 50% of their clinical time evaluating possible child abuse cases including physical abuse, and have access to an Institutional Review Board. CAP board certification became available in 2009 after participant recruitment. Participants submitted demographic information about themselves including sex, race and ethnicity, and years in practice.

### Study procedures

Each CAP submitted completed consultation notes using a secure, web-based interface every 3 months (quarterly). Only completed consultations, for which all examination results were available and the CAP had reached a diagnosis, were requested to insure that the study procedures did not influence clinical decision making. Consultations were selected based on randomly generated dates for each injury type starting in the previous quarter and including a 90 day window to reduce selection bias. CAPs were instructed to select consultations that occurred on or closest to the random date and the instructions specified whether the CAP should select consultations sequentially that occurred early or later in time from the random date. CAPs submitted five cases (two neurotrauma, two long-bone fracture, and on skull fracture) in each submission cycle dependent on availability of the case types. Injury types were limited to neurotrauma, long bone fracture or skull fracture in children up to 4 years of age. The web-based interface prompted CAPs to enter their note in a standard medical format including the history of presenting illness, past medical history, review of systems, family history, social history and physical exam.

#### Child race, ethnicity, and SES

For each case, CAPs recorded demographic information including child age, sex, and insurance type. Perceived race and ethnicity were noted. Physicians were asked to rate perceived family SES using a sliding scale of 1 = low to 100 = high.

#### Social risk perception

CAPs ranked their perception of the social risk of abuse for the child (perceived social risk) using a visual analogue slider scale anchored at 1 = low and 100 = high. To identify factors contributing to risk perception, content analysis was used to extract three categories of elements from the text of the consultation note: risk indicators, social cues and non-social cues [6]. *Risk indicators* were defined a priori as risks for child abuse identified in population based outcome studies and were grouped into four categories: family, parent, child, and social risks [7–9]. *Social cues* developed de novo were defined as comments in the consultation note reflecting the positive and negative social ecology of the child (e.g. parents attend church weekly) and included CAP perceptions of the family (e.g. mother and father appear appropriately concerned). *Non-social cues*, developed de novo, were defined as factual pieces of information recorded in check-list fashion that could reflect positively or negatively on the family (e.g. child’s vaccinations are up-to-date) Table 1.

**Table 1:** Categorization of risk indicators, non-social and social cues in child abuse pediatrician notes (*n* = 730)

Population based risk indicators	
Child risks	Low birth weight or premature
	Disabled or behavioral disorder
Family risks	Single mother
	Re-ordered family
	Intimate partner violence
Parental	Young maternal age
	Substance abuse
	Psychiatric illness
	Low educational achievement
Social	Unemployment
	Poverty
	Social isolation
Non-Social Cues	
Negative	Immunizations not up to date
	Gas or colic drops (crying is associated with abusive head trauma)
	Unplanned pregnancy
	Do not follow parenting guidelines (e.g.car seats)
	Late/inconsistent prenatal care
	Missing well child care visits
	No primary care provider
Positive	Primary care provider noted
	Employment for either caregiver
	Immunizations are up to date
	Follows parenting guidelines (e.g. uses car seats)
	Well child care visits attended
	Consistent, early prenatal care
Social Cues	
Negative	Negative description of male caregiver
	Negative description of female caregiver
	Prior CPS involvement in the family
	Risk family situation. Household described as chaotic, dangerous, or dysfunctional.
	Caregiver with criminal justice history (arrest, probation, parole, incarceration)
	Changing history/ blame shifting
	Caregiver delayed care for current injuries
	Incompetent caregiving
	Caregiver inferred mental health problems
	Caregiver’s own abuse experience
	Prior trauma history for patient
	Inferred substance abuse
	Intimate partner violence (prior family)
	Caregiver negative description of child
Positive	Sought appropriate care for current injury
	Caregivers positive description of child
	Social support available
	Competent parenting
	Positive description of male caregiver
	Sought non-emergent care in the past
	Positive description of female caregiver
	Caregivers are professionals
	Positive description of family
	Provide thoughtful child care

#### Scale construct validation

Construct validity was assessed for the perceived risk, SES and certainty scales in a small pilot study. As expected, perceived risk was higher in cases of abuse and reduced in a stepwise fashion for intermediate and not abuse determinations (*p* < 0.001). Perceived risk was similar when CAPs had similar amounts of information but decreased when social cues were removed (*p* = 0.07). Perceived SES was compared to insurance status. Higher SES ratings were correlated with private insurance (means: 55.3 private insurance, 38.1 public insurance, *p* = 0.04). Finally, among cases with severe injuries and no plausible mechanism for injury other than abuse, certainty was high among CAPs with and without social information (means 94.5 verus 95.3, respectively, *p* = 0.98) although perceived risk dropped (means 66.8 versus 28.0 for CAPs with and without social information, respectively, *p* = 0.12) showing that certainty acted independently of perceived risk.

#### Diagnostic decision-making

At the conclusion of each case, the CAP recorded his or her diagnosis: probable abuse, probable not abuse, or indeterminate. CAPs rated how certain they were that their diagnosis was correct on a scale of 1 (not certain) to 100 (very certain). Certainty was not rated for indeterminate cases.

#### Reversed cases

To confirm that perceived risk was associated with social risk as written in the notes, the first two cycles of submitted cases without an indeterminate diagnosis and with either high (≥ 75) or low (≤ 40) social risk as rated by the CAP who entered the case, and whose certainty was 90% or less were reversed. The reverse methods were created for this project to account for more subtle social factors than SES alone included in the consultation notes that may affect CAP risk perception. Certainty of 90% or less was chosen to exclude cases in which there was a confession, witness or no other potential injury mechanism than abuse. To reverse cases, all social risk indicators and cues were removed from the case and replaced with opposing risk indicators and cues that had been identified during content analysis. For example, a consultation note that stated that mother was employed at a convenience store, that she lacked social support, and had received treatment for depression as a teenager would have these elements removed and systematically replaced with a higher level of employment such as accountant or librarian, social support from a friend or family member, and no mention of prior mental illness. No information on the child’s race or ethnicity was included to remove this as a potential variable. A second investigator reviewed all of the reversed cases to ensure that the process was systematic and the meaning of the case and injury mechanism remained intact. The physical exam findings, past medical history and laboratory and radiographic findings were not altered. Participating CAPs naïve to the original cases and chosen at random rated each case as written (without race or ethnicity information) or the reversed cases. Similar to the original cases, CAPs rated social risk, their diagnosis, and their level of certainty with the diagnosis.

### Analysis

#### Qualitative analysis

Two investigators reviewed the first 100 notes independently to define basic codes, select descriptive text and develop a data dictionary. Each note was discussed and disagreements were resolved by consensus. Basic codes were grouped into categories under higher-order headings that mapped to risk indicators, social cues and non-social cues. One investigator coded the remaining cases and the second investigator recoded every 10th case to prevent coding drift [10]. Disagreements prompted review of prior cases and categories to ensure that both investigators agreed to the application of definitions in all cases [11].

#### Quantitative analysis

Physician race and ethnicity were categorized as non-minority (Caucasian or Asian) or under-represented minority. Asian Americans are not considered under-represented in medicine in the United States [12]. Injury case demographics were described including child age, sex, race and ethnicity, and injury type. Child race and ethnicity were categorized into minority and non-minority as above. Asian American children were included with non-minority children because Asian Americans may be perceived as “model minorities” in American culture [13]. Case characteristics included the range and median of continuous variables, and the counts of risk indicators, social cues and non-social cues. The percent of reverse cases that changed diagnostic category (probable abuse, indeterminate, probably not abuse) were calculated. Bivariate analysis described the associations of specific social cues with perceived risk, and the association of certainty with perceived risk. Certainty and perceived risk were stratified by probable abuse and probable not abuse diagnoses.

#### Mixed methods analysis

**Fig. 1:**
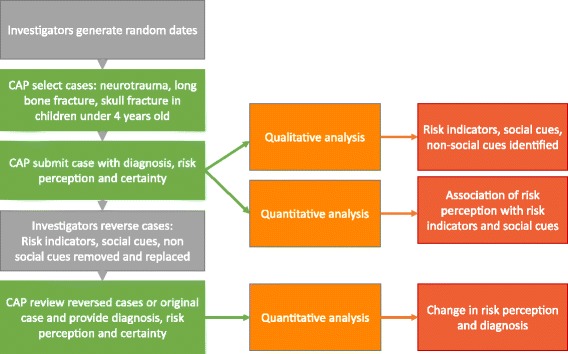
Relationship of study procedures to mixed-methods analysis

The relationship of study procedures to the mixed-methods analysis is shown in Fig. 1. A linear mixed-effects model was used to examine associations of child minority status and perceived SES with social risk perception after adjusting for risk indicators, social cue and non-social cue counts. To account for clustering of cases by physician and by physicians within centers, a random effect for physician and for clinical center was included in the model. The center covariate was removed as it was not statistically important (*p*-value >0.2). Covariates in the model included physician and child demographics, perceived SES, minority status and injury type. Perceived SES and race/ethnicity were collinear in the model. Thus, a race/SES variable was created by dividing perceived SES into tertiles (low, middle and high) for both the minority and non-minority groups creating a race/SES variable.

Forty-seven (6.4%) cases were missing race and ethnicity information. Complete information was observed in all other analytical variables. To account for these missing data, we used multiple imputation with chained regressions, as implemented in IVEware, for each of ten imputed data sets. Studies comparing multiple imputation to listwise deletion methods show that imputation produces less biased odds ratio estimates [14]. Multiple imputation analyses were conducted on the ten imputed data set and the results combined using the SAS procedure PROC MIANALYZE.

## Results

### Demographics

The 32 physician participants from 23 institutions contributed 746 consultation notes for children referred for suspected physical abuse. Sixteen cases were excluded for age over 4 years or incorrect injury type leaving 730 cases for analysis. Physicians were experienced (63% with 10 years or more experience), primarily female (84%), and non-minority (84%). Children had a median age of 7 months (IQR: 3–13 months), were majority male (58.5%), publicly insured (69.2%), with 49.4% minorities. Injury types were divided into neurotrauma (33%), long bone fracture (39%) and skull fracture (28%).

### Risk indicators, social and non-social cues

Most cases (64.7%) had at least one risk indicator reflecting published, population-based abuse risk in child (14.2%), family (40.4%), parental (21.5%), and social (27.3%) categories. Most cases included at least one negative (64.8%) and positive social cue (59.6%). Positive non-social cues were included in 85.1% of cases; and, 26.7% of cases contained negative non-social cues.

### Perceived social risk

The median perceived social risk was 59 (IQR: 30, 75), range 1–100, and the median perceived SES was 35 (IQR 22, 50), range 1–100. In the unadjusted analysis, significant demographic associations with perceived risk included minority physician status and child injury type. Minority physicians perceived significantly lower mean social risk compared to non-minority physicians. Perceived social risk was higher for children with neurotrauma compared to skull fracture (Table 2).

**Table 2:** Unadjusted and adjusted estimates of physicians’ perception of social risk

Covariate	Unadjusted		Adjusted Model	
	Perceived Risk Estimate	95% Confidence Interval	Perceived Risk Estimate	95% Confidence Interval
Physician Characteristics				
Experience (<10 years)	5.0	−2.0, 11.9	0.7	−7.2, 8.6
Sex (Female)	1.3	−8.4, 10.9	−5.8	−16.4, 4.7
Race/Ethnicity (minority)	−11.3	−20.3, −2.3	−12.5	−23.4, −1.8
Child Characteristics				
Age (Months)	−0.1	−0.3, 0.1	−0.2	−0.3, −0.03
Sex (Female)	−4.6	−8.5, −0.7	−3.4	−6.3, −0.5
Injury Characteristics				
Neurotrauma	12.5	7.7, 17.3	7.0	3.2, 10.7
Extremity Fracture	3.5	−1.1, 8.2	0.6	−3.0, 4.1
Skull Fracture	referent		referent	
Child Race/Ethnicity and Perceived Family SES				
Minority, Low SES	29.2	23.6, 34.8	17.9	12.8, 23.0
Minority, Mid SES	20.6	15.1, 26.1	13.1	8.2, 18.1
Minority, High SES	6.1	−0.2, 12.5	4.1	−1.4, 9.7
Non-minority, Low SES	39.5	32.1, 44.8	24.9	19.2, 30.6
Non-minority, Mid SES	21.9	15.9, 27.5	11.6	6.6, 16.6
Non-minority, High SES	referent		referent	
Risk Indicators Present (Yes)				
Social	15.9	11.6, 20.1	3.9	0.4, 7.5
Family	15.4	11.6, 19.1	5.5	2.3, 8.7
Parent	19.2	14.6, 23.8	6.7	2.8, 10.6
Child	3.2	−2.3, 8.7	0.1	−4.1, 4.2
Negative Social Cue (Number)				
One Cue	8.5	4.0, 13.0	5.3	1.3, 9.4
Two Cues	21.0	15.9, 26.0	13.2	8.6, 17.8
Three or Four Cues	28.7	23.8, 33.6	19.5	15.0, 24.1
Five or Greater Cues	39.2	33.6, 44.8	23.1	17.5, 28.7
Positive Social Cue (Number)				
One Cue	−8.4	−12.7, −4.0	−1.2	−4.7, 2.3
Two Cues	−9.5	−15.5, −3.4	−4.6	−9.4, 0.3
Three or Four Cues	−13.3	−20.1, −6.5	−4.4	−9.9, 1.1
Five or Greater Cues	−36.6	−49.0, −24.2	−21.3	−31.2, −11.4
Negative Non-social Cue (Number)				
One Cue	7.5	2.7, 12.3	−0.5	−4.3, 3.3
Two or More	9.5	1.8, 17.3	3.1	−2.9, 9.0
Positive Non-social Cue (Number)				
One Cue	−14.0	−20.2, −7.7	−9.5	−14.3, −4.7
Two Cues	−13.7	−20.2, −7.1	−7.8	−12.9, −2.7
Three Cues	−13.1	−20.0, −6.2	−6.8	−12.3, −1.3
Four or Greater Cues	−14.6	−21.5, −7.6	−3.7	−9.4, 2.0

Negative numbers indicate lower perceived social risk while positive numbers reflect a higher perceived social risk compared to referent value for each covariate

*SES* Socioeconomic status

The two lowest SES tertiles were associated with higher perceived social risk for both minority and non-minority families (Table 2). Perceived SES was lower for minority families compared to non-minority families (median 30, IQR: 20–45.5 versus median 40, IQR: 25–58.7, *p* < 0.001). Consistent with this perception, minority children were less likely to have private insurance than non-minority children (9.4% versus 32.2%, *p* < 0.001, respectively).

**Fig. 2:**
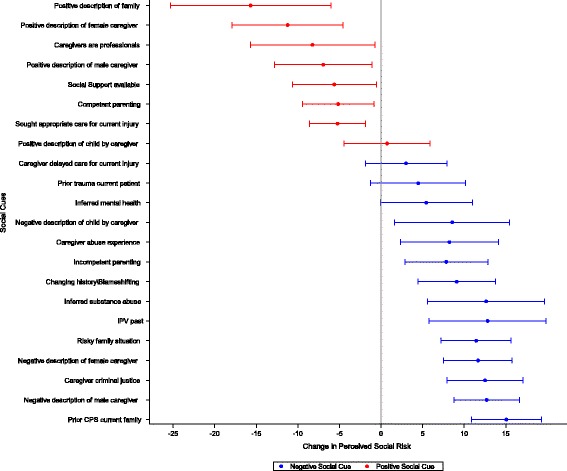
Associations of social cues found in consultation notes with child abuse physicians' perceived social risk

The unadjusted associations of specific social cues and perceived social risk are shown in Fig. 2. A positive description of the family in the consultation note was associated with the lowest perceived social risk (−15.7 points, 95% CI -25.3 to −6.0) while a note about prior family CPS involvement was associated with the highest perceived social risk (+15.1 points, 95% CI: +10.9 to +19.3). Counts of risk indicators, negative and positive social cues, and negative and positive non-social cues were associated with perceived social risk (Table 2).

In the adjusted analysis examining associations with perceived social risk, minority physicians continued to perceive lower social risk compared to non-minority physicians. Perceived social risk was highest for children in the lowest SES categories but differed by minority status. Perceived social risk was highest for non-minority, low SES children (24.9 points, 95%CI: 19.2, 30.6) followed by minority, low SES and middle SES children. Perceived social risk increased with increased numbers of negative cues and decreased with the number of positive social and non-social cues (Table 2).

### Certainty of diagnosis

The median level of diagnostic certainty among physicians was 90 (IQR: 78, 97). Certainty was higher in cases diagnosed as probable abuse (median 95, IQR: 86, 100) compared to probable not abuse (median 85, IQR 80, 90; *p* < 0.0001). Certainty and perceived social risk were associated: in probable abuse cases certainty increased as social risk perception increased (Spearman correlation 0.21, *p* < 0.001) and in probable non-abuse cases certainty decreased as risk perception increased (Spearman correlation (−)0.19, *p* = 0.003).

### Reversed cases

**Fig. 3:**
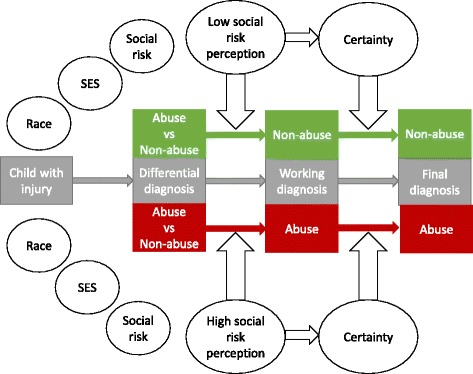
Theoretical model showing the potential pathway of risk perception on diagnostic decision making

When cases were reversed, perceived social risk changed in the expected direction supporting that the measure reflected written social factors. When reversed, perceived risk for high social risk cases decreased from a mean of 82.2 to 30 (mean change 52.2 points, 95%CI: 43.1, 61.3) and the perceived risk for the reversed low risk cases increased from 23.4 to 65.7 (mean change of 42.3 points, 95%CI: 36.3, 48.3). Reversing social risk perception changed the diagnostic decision in 40.2% (95%CI: 30.6, 50.4) of cases. The diagnosis changed from probable abuse to probable non-abuse (or vice-versa) in 12.7% of cases; from indeterminate to probable abuse or probable not abuse in 16.7% of cases; and from probable abuse or not abuse to indeterminate in 10.8% of cases. Figure 3 displays a model of how risk perception may influence diagnostic decision making through certainty.

## Discussion

This study found that the CAP perception of the child’s SES is strongly and independently linked to physician perception of risk for abuse after adjustment for physician, child and family characteristics. While the relationship of SES to differential child abuse evaluation has been described previously in non-CAP physicians [1, 4], this study shows that SES plays a role in CAP perceptions of abuse risk. The experimental model in this study that reversed social risks, demonstrates that CAP social risk perception is associated with diagnostic decision-making.

CAP risk perception for abuse encompasses child, family, parental and social risk indicators; social cues reflecting CAP impressions of the caregivers and their actions; and, non-social cues such as attendance at well child visits. After taking these factors into account, CAP perceptions of risk remained independently associated with the child’s perceived SES. While child abuse may be more prevalent in poor communities, population-based studies show that the attributable risk of poverty to abuse is low, [8] that is, most poor families do not abuse their children. Laskey’s study found that general pediatricians are more willing to consider an abuse diagnosis among poor children [1]. Our findings add to Laskey’s work by using clinical cases rather than scenarios, adjusting for multiple aspects of the social history, and quantifying physicians’ perceptions of SES. Prior studies have stratified patient SES based on public/private insurance status which provides little differentiation in this largely publicly insured population. Asking physicians to quantify their perception of SES allowed us to both stratify a race/SES association and to link their perception of SES to social risk.

CAP risk perception is important because it may unconsciously influence diagnostic decision-making. We changed CAP risk perception by reversing social elements of the cases while leaving the injury and medical history and findings intact. Reversal of social risk was associated with a change in CAP diagnosis in 40% of cases. This is not an insignificant change in diagnosis. In fact, the change in diagnostic decision from reversing social risk is a more significant change in diagnostic decisions than has been documented with follow-up skeletal surveys, a strongly recommended diagnostic test in the evaluation of child physical abuse [15, 16].

Our theoretical model proposes that risk perception may act through CAP diagnostic certainty. Certainty or confidence in diagnosis has been linked to common cognitive errors, such as biased evidence gathering, or failing to incorporate discrepant information that does not support a diagnosis (anchoring) [17]. High social risk in abuse cases is consistent with CAP expectations leading to certainty, but not necessarily leading to diagnostic accuracy [18]. The relationship of risk perception to certainty is a potential explanation for under-diagnosis of abuse in higher SES families (where risk perception is low leading to low certainty in the diagnosis of probably abuse) as documented by Jenny, and increased comfort for reporting abuse in low-income families (where risk perception is high leading to higher certainty for diagnosis of probable abuse) shown by Laskey. As minority families are disproportionately low income in the United States, this would lead to a differential evaluation of minority children evaluated for abuse as has been noted in the literature [19]. Our data support another potential role for risk perception. Risk perception may influence CAP concern about the consequences of misdiagnosis for children and families. Implicitly or explicitly, CAPs may weight concerns of a missed diagnosis of abuse more heavily than an incorrect diagnosis of abuse in cases with high risk perception and weight the incorrect diagnosis of abuse more heavily in cases with low risk perception.

If diagnostic decision-making is influenced by risk perception, it may be possible to correct this step of the diagnostic process. Some authors suggest that switching from an intuitive to analytic thinking process through the use of reflection or decision support tools, such as checklists may ‘de-bias’ decisions [20]. A small single center study that introduced specific guidelines to guide the medical evaluation of suspected abuse among head injured children reduced racial disparity in these evaluations [21]. This suggests that explicit protocols for clinical decision-making may reduce differential decision-making.

There are important limitations to this research. We made the assumption that physicians documented the major social risks that they felt were important to formulate perceived risk. If our assumption was incorrect, it is possible that the race/SES association is incorrect. Our assumption was supported by the change in perceived risk with the numbers and types of risk indicators and cues identified in the qualitative analysis. Additionally, prior studies reporting physicians’ results on implicit association tests have found that Caucasian physicians, like the general population, show an implicit preference for Caucasians, and African American physicians, as a group, do not show a preference [22]. This is consistent with our results. We did not perform inter- or intra-rater reliability of our scales; however, we did perform construct validity testing. We studied CAPs who were interested in participating in this project not a random sample. Participating CAPs may differ from the pool of all CAPs. There is likely an underlying rate of diagnostic disagreement between CAPs even when given the same information that is not taken into account in our analysis. To examine this, we performed a separate analysis that found that the CAP who saw the child and submitted the case disagreed with the CAPs who reviewed the case notes as written in 11% of the probable abuse cases with less than 2% changing from probable abuse to not abuse; 23% of the probable not abuse case changed, with a 2.6% change to probable abuse. This adds strength to our argument that the 40% change in diagnosis is linked to the change in risk perception. Finally, the number of minority physician participants in this study was low; thus, analyses of physician race should be interpreted cautiously. Strengths of this study include the national sample of CAPs, our ability to measure CAPs perceptions of risk and SES, to consider more nuanced social information than is found in administrative datasets, and to link a potential mechanism to the disparities in diagnosis.

## Conclusions

CAPs have different perceptions of child abuse risk based on the child’s social ecology. Poor children are perceived to be at the highest risk for abuse independent of other social factors. Perceptions of social risk appear to shape diagnostic decision-making by influencing physician certainty related to a diagnosis of abuse. Our findings help to explain the differential decision- making for poor and minority children reported in the literature. Future research is warranted on the use of explicit decision-making tools to reduce this noted disparity.

## Abbreviations

CAP: Child abuse pediatricians
CI: Confidence interval
IQR: Interquartile range
SES: Socioeconomic status
